# Distribution of trace elements in benthic infralittoral organisms from the western Antarctic Peninsula reveals no latitudinal gradient of pollution

**DOI:** 10.1038/s41598-021-95681-5

**Published:** 2021-08-11

**Authors:** Paula De Castro-Fernández, Luis Cardona, Conxita Avila

**Affiliations:** Department of Evolutionary Biology, Ecology and Environmental Sciences & Biodiversity Research Institute (IRBio), University of Barcelona, Diagonal Ave. 643, 08028 Barcelona, Catalonia Spain

**Keywords:** Environmental impact, Food webs, Environmental monitoring, Metals

## Abstract

Antarctica is considered one of the most pristine regions on Earth, but evidences of global and local anthropogenic pollution exist. Chromium (Cr), lead (Pb) and mercury (Hg) are bioaccumulated and sometimes biomagnified through the trophic web. We aim to determine whether a latitudinal gradient of these trace elements exists in benthic organisms along the rocky shores of the Antarctic Peninsula and the South Shetland Islands. Levels of Cr, Pb, and Hg were measured by ICP-MS in two macroalgae (*Palmaria decipiens* and *Desmarestia anceps* or *Desmarestia menziesii*), one gastropod (*Nacella concinna*), two starfishes (*Odontaster validus* and *Diplasterias brucei*), and suspended particulate organic matter (SPOM) from five sampling sites ranging in latitude from 62°11′17″S to 67°33′47″S. Levels of trace elements differed among sites and species, but no latitudinal gradient was observed for these pollutants. Levels of Hg and Pb in animals were consistent with biomagnifications along the food web, as were higher is starfish than in limpets. However, macroalgae and SPOM are unlikely to be the main primary producers supporting those consumers, as Hg levels in macroalgae and Pb levels in SPOM were much higher than in animals. The levels of trace elements detected were similar or higher than in other Antarctic places and other regions of the world, thus indicating that the Antarctic Peninsula area is as polluted as the rest of the world.

## Introduction

Trace elements current average concentration is less than about 100 parts per million atoms (ppma) or less than 100 μg g^−1^^1^. They occur naturally in the earth crust, being present in soil parent materials and in the surface soil in variable proportions^2–4^, but human activities, mainly mining, metal processing, fossil fuel combustion, use of pesticides, and waste disposal have introduced high quantities of them in the environment, thus resulting in significant pollution levels^2,3,5–7^.

Antarctica is thought to be one of the last untouched and wild areas on Earth, since its remote location and the only recent, scarce, and highly seasonal human presence^8^. Nevertheless, there has been evidence of diverse anthropogenic impacts for a long time^9,10^. Anthropogenic trace element pollution in Antarctica may either be the result of global pollution or be produced locally^11,12^. The arrival with marine currents is largely constrained by the broad belt of the Southern Ocean’s water, namely the Antarctic Circumpolar Current, constituting a barrier to this transport^13^. Thus, trace elements from other continents, primarily those in the Southern Hemisphere, are mainly transported with air masses that move towards Antarctica in what is called the long-range atmospheric transport (LRAT)^13–16^.

Recently, more attention has been paid to the contaminants emitted locally in Antarctica as a result of increasing human activity^17^. As early as 1987, it was determined that four-fifths of total Pb in Antarctic air at that time had an anthropogenic origin^18^. Later, it was observed that levels of some trace elements (such as Pb and Zn) in the atmosphere over the Antarctic Peninsula were higher than it would be expected due to aerosol contribution^19^. Anthropogenic local pollution could be an explanation and local contamination caused by research stations and their associated activities, such as ship operations, sewage production, fuel consumption, and waste disposal, are the major sources of local contamination, as well as the developing tourism industry^16,20,21^. The Antarctic Peninsula and the South Shetland Islands are the most vulnerable regions to local pollution because they concentrate most of the human activity in the continent^8,12,22–24^. Accordingly, Jerez et al.^25^ reported a latitudinal gradient in the levels of Pb, Cr, Al, and Mn in feathers of Adélie penguins *Pygoscelis adeliae* nesting along the Antarctic Peninsula. However, such pattern might not exist for species inhabiting shallow benthic habitats, more influenced by local processes. Currently, there is a wealth of studies reporting the levels of trace elements in Antarctic, benthic organisms^11,26–29^, but we are not aware of any study addressing the existence of a latitudinal gradient in the levels of any trace element in benthic species.

Mercury (Hg) and lead (Pb) are non-essential elements that are toxic even at low concentrations^30^. They may displace other elements that act as enzymes cofactors and therefore diminish or block physiological processes^31^. Chromium (Cr) is an essential trace nutrient element that can be toxic at high concentrations^32^, for example causing impairment of photosynthetic energetic pathway processes, blocking cell division or inhibiting enzyme activity in microalgal cells^33^. Hg, Pb and Cr are among the most relevant anthropogenic trace pollutants in Antarctica. Indeed, Pb and Hg are considered priority pollutants and Hg is also classified as a priority hazardous substance by the regulation in force^34^. Hence, it is important to monitor the concentration of these—and other—elements at different sites in Antarctica as they are highly related to human activities^25,35^. Furthermore, assessing the concentrations and effects of trace elements on Antarctic marine organisms could contribute to prevent the loss of ecosystem services that Antarctic biodiversity provide^36^. The choice of these elements is further justified since most invertebrates are not capable of regulating their body concentrations^37–39^. Therefore, the levels of these elements in the organisms represent or correlate to the levels in the environment. Hg is particularly interesting because it biomagnifies through the food chain and may be therefore useful as trophic tracer^40^.

Sediment conditions usually represent the average state of the system as they have high physicochemical stability^41,42^. They can act as trace elements or pollutants reservoirs, offering a history of pollution of the environment^43–45^. Also, trace elements concentration in water masses could be extrapolated from sediments analysis^23^. Therefore, determining trace element contamination in sediments could provide information about marine environment conditions and represent average water quality^46^. Nevertheless, some studies concluded that measuring the concentration of a chemical in the organism is more useful to predict the effects of the substance in the organism, i.e. toxicity, than analyzing environmental levels such as sediment or water concentration. Marine organisms incorporate trace elements from the environment and accumulate some of them in their soft tissues, where levels of contaminants are several orders of magnitude above the environmental levels^20^. One advantage of using body concentration as an indicator of bioavailability is that environmental chemical conditions, such as salinity, pH, or temperature, as well as the chemical state of the element, which may affect the element toxicity, can be avoided^47^. Besides, this measure integrates the accumulation of the chemical due to exposures that may be intermittent, from different origins and different compartments^48^. However, the element must not be regulated by the organism for body concentration to be a good indicator of toxic effects. Thus, the level of the element in the organism must increase with increasing environmental concentrations^24^.

In addition to bioaccumulation, some elements biomagnify trough the food chains, i.e., they are transferred to higher trophic levels via dietary uptake^49^. As a consequence, organisms at higher trophic levels have higher body levels of those elements than their prey, which makes them useful as trophic tracers. It should be noted, however, that Antarctic benthic animals rely on a diversity of primary producers, including sympagic algae, microphytobenthos, macroalgae, and phytoplankton^50–54^, although phytoplankton dynamics plays a major role in structuring the benthic marine food web of the region^55^.

The main aim of this study is to assess and compare the levels of the three trace elements (Cr, Pb, and Hg) in suspended particulate organic matter and five benthic species from shallow, sheltered rocky bottom ecosystems along a latitudinal gradient along the South Shetland Islands and the western Antarctic Peninsula to test the hypothesis that the pollution levels decrease southward in parallel to human activity. The selected species included three primary producers, one herbivore and two carnivores^55^.

## Materials and methods

### Study area

**Figure 1:**
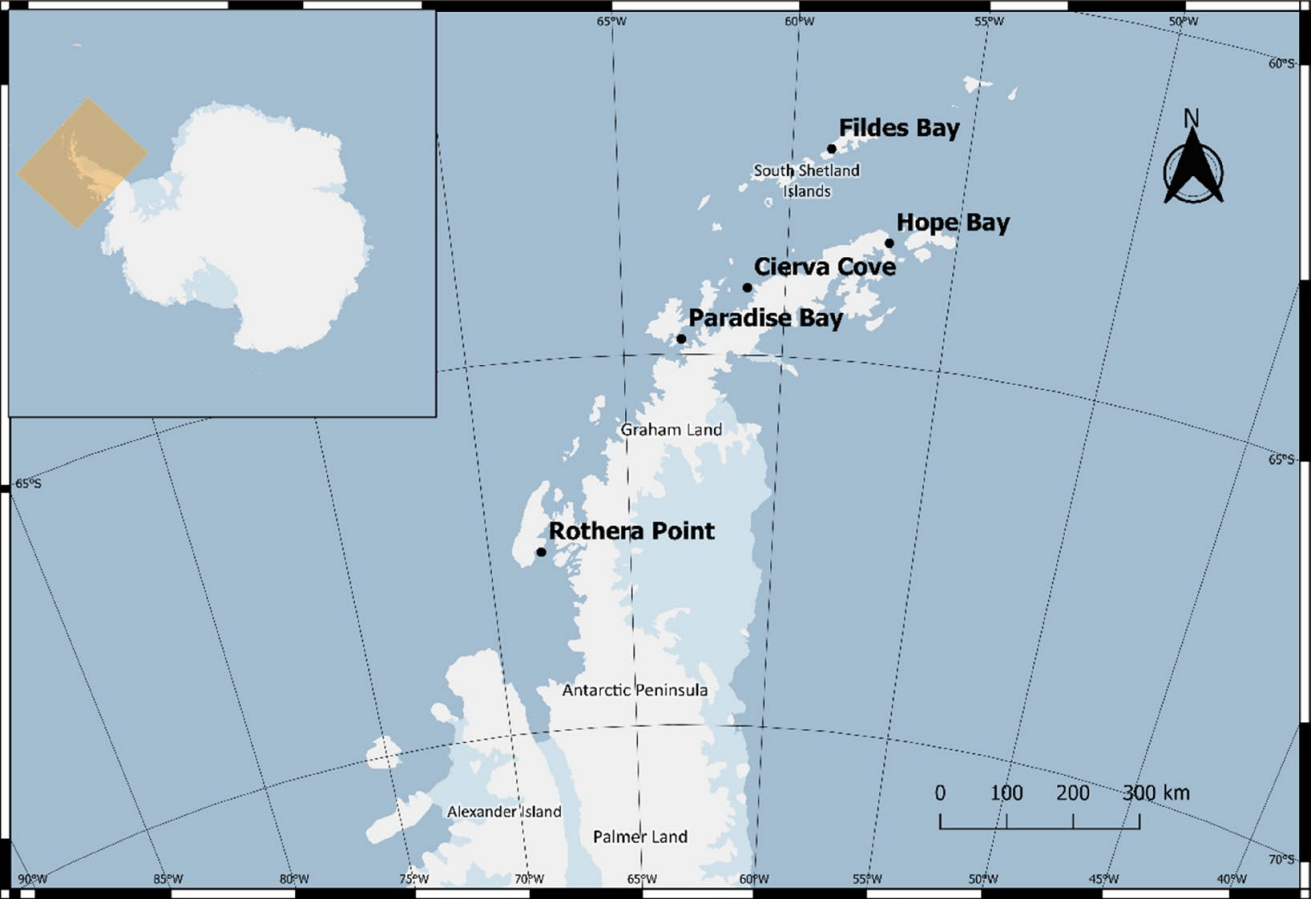
Study area and location of the sampling sites. The map was created using the Free and Open Source QGIS 3.16 software (http://www.qgis.org/) and the Quantarctica 3.2 project (https://www.npolar.no/quantarctica/).

Five sites along the South Shetland Islands and the Antarctic Peninsula were chosen for the study: Fildes Bay (King George Island), Hope Bay, Cierva Cove, Paradise Bay, and Rothera Point (Fig. 1). These locations cover a latitude from 62°12′7″S in Fildes Bay to 67°34′30″S in Rothera Point, and hence most of the Antarctic Peninsula latitudinal extension. They provide a large enough extension to test the existence of a latitudinal gradient in trace elements pollution. All stations are located in closed and sheltered coastal zones and hence comprise highly sensitive local conditions. Furthermore, all the selected locations are situated near human facilities, mainly research stations^56^, which are the most likely sources of local pollution.

### Species selection and sampling

Subtidal, sheltered rocky bottoms around Antarctica share a similar community, dominated by the canopy forming brown macroalgae *Desmarestia anceps* and *Desmarestia menziesii* and a dense undergrowth of the red macroalga *Palmaria decipiens*^57^. *Desmarestia menziesii* is usually the dominant species just below the heavy scour area, being eventually replaced at greater depths by *Desmarestia anceps*^57^. However, there are locations where only one of these two *Desmarestia* species are present^57^. The starfish *Odontaster validus* is also widespread around the whole continent^58^ and the starfish *Diplasterias brucei* is also thought to be circumantarctic, although detailed information is missing^58^. Both are very abundant in shallow kelp forests off the western Antarctic Peninsula. Finally, the limpet *Nacella concinna* is restricted to the western Antarctic Peninsula^59^, where it is one of the most conspicuous gastropods from the shallow kelp forests. Stable isotope analysis has confirmed that *Nacella concinna* is an herbivore and the two starfish species are carnivores^55^.

Five specimens of each of species were collected at five different sites along the South Shetland Islands and the western Antarctic Peninsula during the DISTANTCOM cruise (CTM2013-42667/ANT) from February 12th to February 22nd, 2016 (Table 1). A sample size of five or less is extensively used to determine the concentration of trace elements in marine communities^20,26,27,29,60^.

**Table 1:** Taxa, number and sites of collection of the samples.

Phylum	Species	Location				
		Fildes Bay	Hope Bay	Cierva Cove	Paradise Bay	Rothera Point
		62°11′17.3″S	63°22′18.4″S	64°05′26.3″S	64°53′43.7″S	67°33′47″S
		58°52′16.8″W	56°58′55.7″W	60°59′06.7″W	62°55′48″W	68°10′01.6″W
Several	(SPOM)	5	5	5	5	5
Ochrophyta	*Desmarestia anceps *Montagne, 1842	0	5	5	5	5
	*Desmarestia menziesii *J.Agardh, 1848	5	0	0	0	0
Rhodophyta	*Palmaria decipiens* (Reinsch) R.W.Ricker, 1987	5	5	5	5	5
Mollusca	*Nacella concinna* (Strebel, 1908)	5	5	5	5	5
Echinodermata	*Diplasterias brucei* (Koehler, 1907)	5	5	5	5	5
	*Odontaster validus* Koehler, 1906	5	5	5	5	5

*SPOM* Suspended Particulate Organic Matter.

Benthic organisms were collected by SCUBA diving at depths between 10 and 15 m. Organisms were detached from the rocky bottom by hand or with the aid of a knife and were placed in 1 L clean plastic containers. Suspended particulate organic matter (SPOM) was also collected at the five sampling sites, as sinking phytoplankton plays major structuring the food web of shallow kelp forests^55^. SPOM was collected with a 50 μm mesh size plankton net towed horizontally at 5 m depth at low speed (1.85–3.7 km/h). Each SPOM sample is the result of towing the plankton net for 4–10 min, depending on plankton density at each site.

### Sample processing

Subsamples of Suspended Particulate Organic Matter (SPOM) were visually checked under an optical microscope immediately after collection to assess the dominant groups. They consisted in diatoms (Ochrophyta) and dinoflagellates (Myzozoa) in all cases. All samples were frozen at − 20 °C and once in the laboratory at the University of Barcelona (UB), they were thawed on ice and processed prior to analytical determinations. Two ml of concentrated phytoplankton were collected and a 2 × 2 cm fragment of epibiont-free blade of the macroalgae was selected. Limpets were dissected and the gut and its contents removed. Limpet radulas and shells were also discarded. For starfishes, only 1 to 3 arms were sampled to analyze each specimen, after discarding the gut. For trace elements determination, we followed the protocol established in the Scientific and Technological Centers of the UB (CCiTUB, http://www.ccit.ub.edu/EN/), standardized and validated in previous works^60–65^. In these works, the entire analytical procedure was validated by analysing one or more blanks, replicates and one certified reference material for every batch of samples. Replicates were found to differ below 10% and the recovery percentage fell between 90 and 100%^60–65^. Samples were dried for 24 h at 60 °C and then homogenized to powder using a ceramic mortar and pestle. 100 mg dw of each homogenized sample were digested with a 2:1 HNO_3_ (69–70% Baker Instra—Analyzed Reagent) and H_2_O_2_ (30% Suprapur Merck) solution in Teflon vessels previously cleaned with HNO_3_ under pressure at 90 °C for 24 h. For this, 2 mLof HNO_3_ and 1 mL of H_2_O_2_ were used. The digested solution was diluted with 20 mL of ultrapure water (HNO_3_:H_2_O 1:10). Cr, Pb, and Hg were determined in the diluted digested solution by ICP-MS (PerkinElmer NexION 350D). Three digestion blanks were prepared in each sample digestion series (25 samples) to assess contamination during the analytical procedure.

### Data analysis

General linear models (GLM) were used to assess differences in the levels of Cr, Pb, and Hg across species and sites. Previous research has suggested that *Odontaster validus* and *Diplasterias brucei* fed on *Nacella concinna*^55,66^. In order to check this hypothesis and assess the existence of a significant biomagnification pattern, general linear models were performed for each trace element including only these three animal species. A multivariate cluster analysis, using the squared Euclidean distance as a metric and UPGMA as the clustering method, was performed to assess whether the distinct groups of samples recovered according to their levels of Cr, Pb and Hg matched any latitudinal pattern. Furthermore, Principal Component Analysis (PCA) was performed to assess the hypothesis that geographically close sampling sites would lay closer in the space defined by the levels of Cr, Pb and Hg in the five benthic species. The main objective of PCA was to characterize each sampling site by projecting the data in a much smaller sub set of new variables called principal components. To do so, we characterized the benthic community from each sampling site using eighteen variables, corresponding to the levels of each trace element in each species. All the variables were standardized prior to analysis. These new variables extracted by PCA are linear combinations of the initial variables, but highlight the variance within a data set and remove the redundancies, and are orthogonal. Only principal components with an eigenvalue higher than 1 are considered for further analysis^67–69^. General linear models were performed using SPSS Statistics v23 (IBM Corporation) and Principal Component Analysis was performed using PRIMER v7 (PRIMER-e).

## Results

**Figure 2:**
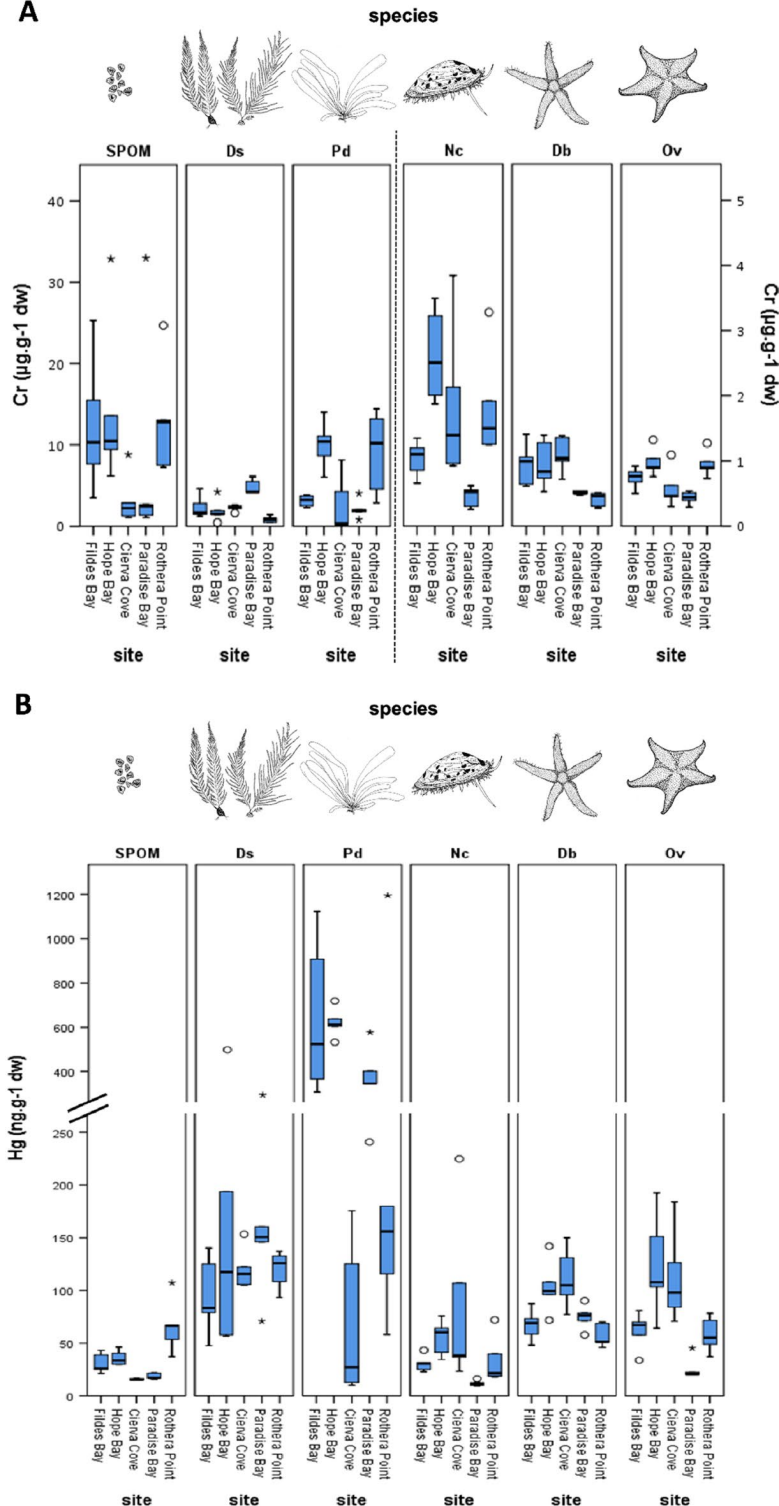
Concentration of Cr and Hg in the different sites and species studied along the Antarctic Peninsula. (**A**) Cr, values expressed as μg g-1 of dry weight. The values of Cr for the primary producers were much higher than the animal species, so they were represented separately and scale changed accordingly. (**B**) Hg, values expressed as ng g^-1^ of dry weight. Discontinuous Y-axis was used to facilitate visualization. Central lines show the mean, boxes extend to the 25th and 75th percentile, whiskers extend to 1.5 × IQR, dots and asterisks are outliers. *SPOM* Suspended Particulate Organic Matter, *Ds* *Desmarestia* spp., *Pd* *Palmaria decipiens*, *Nc* *Nacella concinna*, *Db* *Diplasterias brucei*, *Ov* *Odontaster validus*. The images of species were drawn by the authors of this article.

**Figure 3:**
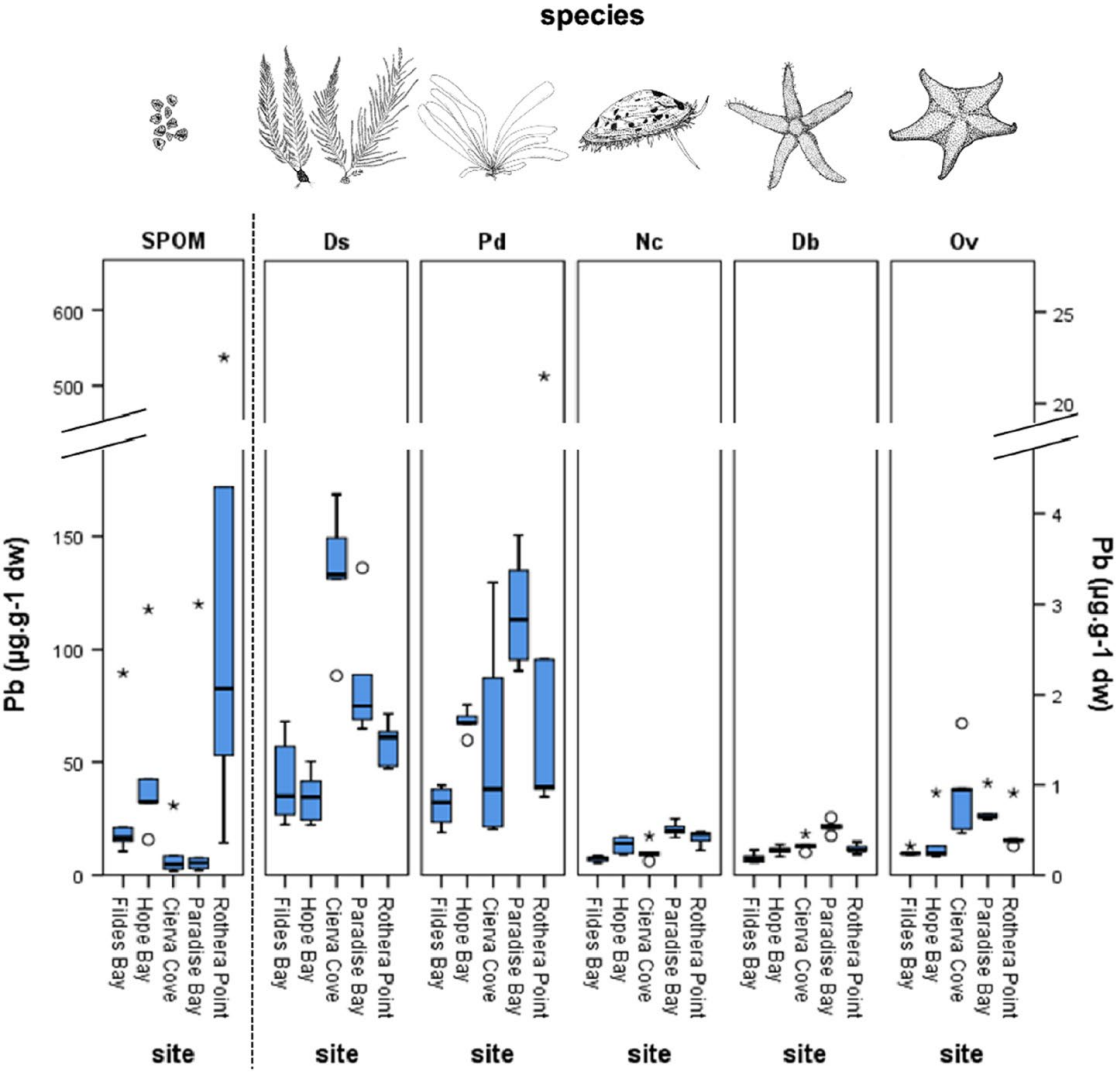
Concentration of Pb in the different sites and species studied. The values of Pb for the SPOM were in different magnitude order than the benthic species, so they were represented separately and scale changed accordingly. Values are expressed as μg g^−1^ of dry weight. Discontinuous Y-axes were used to facilitate visualization. Central lines show the mean, boxes extend to the 25th and 75th percentile, whiskers extend to 1.5 × IQR, dots and asterisks are outliers. *SPOM* Suspended Particulate Organic Matter, *Ds* *Desmarestia* spp., *Pd* *Palmaria decipiens*, *Nc* *Nacella concinna*, *Db* *Diplasterias brucei*, *Ov* *Odontaster validus*. The images of species were drawn by the authors of this article.

Levels of Cr, Hg, and Pb in all the studied species were measured at the five sites (Figs. 2 and 3). The data values represented in Figs. 2 and 3 are provided in Supplementary Table 1. Cr and Pb levels were positively correlated when the whole data set was considered (Pearson correlation; r = 0.464, p < 0.001), but were uncorrelated with the level of Hg (Pearson correlation; Hg vs. Cr p = 0.138; Hg vs. Pb p = 0.339).

Species differed significantly in levels of Cr, Pb, and Hg. Site had a significant effect on Cr and Hg and was in the verge of significance for Pb (Suppl. Tables 2–4). The interaction term was always significant, thus revealing idiosyncratic variations among species across sites.

The levels of trace elements in primary producers (SPOM, *Desmarestia* spp. and *Palmaria decipiens*) were usually higher than in the animal species from the same sampling site, with SPOM and *Palmaria decipiens* values being higher than those of *Desmarestia* spp. (Figs. 2 and 3). This difference was even larger in the case of the Hg values of macroalgae and those of animals (Fig. 2B). SPOM was highly enriched in Pb (Fig. 3), with values ranging from 1.85 to 537.08 ng g^−1^, therefore multiplying the highest value of the other species by a factor of 25. At the same time, SPOM had in general lower Hg values than the sampled species, while both macroalgae (*Palmaria decipiens* and *Desmarestia* spp.) had higher values than the other species, as highlighted before (Fig. 2B).

Regarding the levels of Cr and Pb in consumers, they varied widely across species and zones, as there was a significant (species × site) term in both models (Suppl. Tables 5 and 6). On the contrary, the interaction term of the general linear model for Hg was not significant (Suppl. Table 7), thus revealing that the same pattern was observed in the three animal species across sites, despite differences in the baseline levels among sites. The starfish species had higher levels of Hg than the limpet at all the sites studied. This corroborates that both starfishes are at a higher trophic level than the limpet.

Cluster analysis revealed no latitudinal pattern for any species, as groups always included samples from distant sampling sites (Suppl. Fig. 1–6). For instance, most samples of SPOM, *Desmarestia* spp. and *Nacella concinna* from the five sampling sites clustered together, and samples of *Palmaria decipiens* from Paradise Bay clustered with samples from distant Fildes Bay and Hope Bay, but not with those from nearby Cierva Cove, which in turn clustered with those from distant Rothera Point. Regarding *Diplasterias brucei* and *Odontaster validus*, samples were split in three and four major groups respectively, all them including samples from at least three sites and most including samples from the two most distant sites (Fildes Bay and Rothera Point).

**Figure 4:**
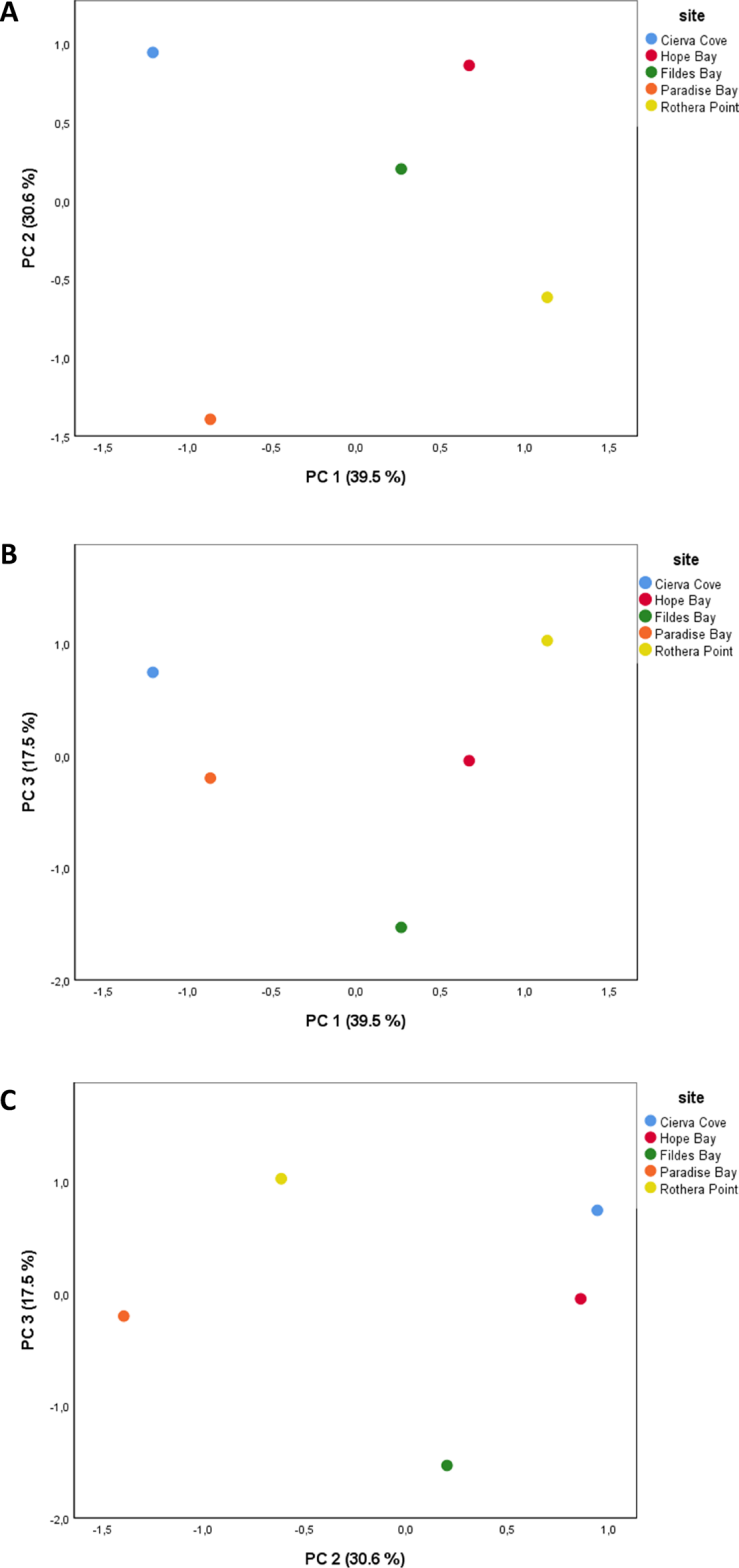
Principal Component Analysis (PCA) score plots. (**A**) PC 1 vs. PC 2. (**B**) PC 1 vs. PC 3. (**C**) PC 2 vs. PC 3.

PCA yielded four principal components with eigenvalues higher than 1 and PC 1, PC 2 and PC 3 explained 87.5% of the variance (Fig. 4). High scores of the first component (PC 1) corresponded to high levels of Cr, Pb, and Hg in SPOM and *Palmaria decipiens* and low levels of these trace elements in *Desmarestia* spp. and the animal species (*Nacella concinna*, *Diplasterias brucei,* and *Odontaster validus*) (Table 2). Contrarily, high scores of the second component (PC 2) were associated to high levels of of Cr and Hg in the animal species and low Pb levels in *Nacella concinna*, *Diplasterias brucei* and both macroalgae (Table 2). High scores of the third component (PC 3) corresponded to high levels of Hg in *Desmarestia* spp. (Table 2). Sampling sites were distributed independently from latitude in the space delimited by PC 1 and PC 2 axes. Certainly, Rothera Point and Paradise Bay had similar PC 2 scores and the same was true for Cierva Cove and Hope Bay, thus supporting the existence of a latitudinal gradient along that axis. However, the northernmost site, Fildes Bay, had intermediate values.

**Table 2:** PCA component matrix. Values represent the contribution of each variable to PC 1 and PC 2.

Variable	Component		
	1	2	3
Cr SPOM	0.920	− 0.068	− 0.248
Pb SPOM	0.763	− 0.335	− 0.243
Hg SPOM	0.897	− 0.183	− 0.223
Cr Ds	− 0.690	− 0.510	0.368
Pb Ds	− 0.840	0.050	− 0.256
Hg Ds	− 0.020	− 0.072	0.981
Cr Pd	0.837	0.211	0.346
Pb Pd	0.427	− 0.604	− 0.103
Hg Pd	0.595	− 0.011	0.330
Cr Nc	0.446	0.734	0.253
Pb Nc	− 0.029	− 0.770	0.461
Hg Nc	− 0.268	0.862	− 0.097
Cr Db	− 0.339	0.897	0.001
Pb Db	− 0.585	− 0.651	0.401
Hg Db	− 0.501	0.731	0.412
Cr Ov	0.907	0.380	0.075
Pb Ov	− 0.806	− 0.051	− 0.013
Hg Ov	0.045	0.943	0.230

*SPOM* Suspended Particulate Organic Matter, *Ds* *Desmarestia* spp., *Pd* *Palmaria decipiens*, *Nc* *Nacella concinna*, *Db* *Diplasterias brucei*, *Ov* *Odontaster validus*.

Cierva Cove and Paradise Bay sampling sites opposed to Rothera Point, Hope Bay and, to a less extent, Fildes Bay along PC 1, and Paradise Bay and Rothera Point opposed to Cierva Cove, Hope Bay and, to a less extent, Fildes Bay in relation to PC 2 (Fig. 4). Fildes Bay and Rothera Point were at opposite ends of PC 3 axis. However, Hope Bay and Paradise Bay had similar PC 3 scores and Cierva Cove was the closest site to Rothera Point along PC 3 axis (Fig. 4). Thus, concentrations of Cr, Pb, and Hg in *Desmarestia* spp. and the three animal species were significantly higher for specimens from Cierva Cove and Paradise Bay, while in Rothera Point, Hope Bay, and Fildes Bay, higher levels of these three trace elements were found in SPOM and *Palmaria decipiens*. In Paradise Bay and Rothera Point, the levels of Pb in *Nacella concinna*, *Diplasterias brucei,* and the two macroalgae were higher than in the other sites. On the other hand, Cierva Cove, Hope Bay, and Fildes Bay showed higher concentrations of Hg and Cr in the animal species than the other sampling zones. Importantly, there was no latitudinal gradient in the ordination of the five sampling sites along any of the two axes.

## Discussion

Levels of Cr, Pb, and Hg in primary producers and consumers from five sampling sites along a latitudinal gradient in the South Shetland Islands and the Antarctic Peninsula revealed no clear latitudinal trend. Instead, the levels of each trace element varied idiosyncratically, as revealed by the significant interaction (species × site) in GLMs. As the species analyzed here belong to the same food web^50–55^, the most plausible explanation to these idiosyncratic changes in trace element burden is that local processes are more relevant for benthic communities than any latitudinal gradient of human disturbance or natural transport of pollutants from areas at lower latitude. Certainly, a latitudinal gradient has been previously reported for the levels of trace elements in the feathers of penguins^25^, but this are pelagic, highly mobile predators, foraging at a much broader scale. On the contrary, the benthic species sampled here were sessile (macroalgae) or had a limited mobility (limpets and starfishes) and hence they seem to be more sensitive to local differences in trace element pollution than to any latitudinal gradient.

Primary producers exhibited the highest levels of trace elements, particularly Cr and Pb, probably because of accumulation mechanisms typical of micro- and macroalgae^70^. Alternatively, Farías et al.^71^ argued that high levels of Cr and Pb in some species may be the consequence of not completely removing fine particulate matter during washing of the specimens after collecting them, although we do not believe this is the case in our study. Even though the trace element content differs among species from the same habitat^72,73^, it has been widely reported that macroalgae concentrate trace elements in their tissues, with concentration factors respect to sea water content as high as 10,000 for Ti and with values of up to 500 for Cr in brown algae, as an example^20,72^. Trace elements can be accumulated in different extra- and intracellular compartments^73–77^. Both micro- and macroalgae take up trace elements and other elements basically through two mechanisms: by attaching them to its cellular surface in a process called biosorption, which is reversible, and by irreversibly binding additional elements after biosorption, often through diffusion to the cytoplasm and binding to proteins or other intracellular structures^70^. Regarding SPOM, its enrichment in trace elements had been reported previously^78^. Diatoms silicic frustule turns to be a highly adsorptive surface that is implicated in the removal of trace element ions from the water column^79^. The attachment of Cr and Pb to diatoms surface would explain the high values reported for these trace elements in the SPOM, since diatoms are the dominant taxa in phytoplankton communities of the inshore waters of the Western Antarctic Peninsula during the summer season^80–83^.

The range and mean values of concentrations of the three elements in our species, together with levels recorded in the literature for the same species from other places in the Antarctic continent are compared here (Table 3). Cr, Pb and Hg levels obtained in the present study can be also compared to those reported in the literature from other regions of the world (Table 4) showing that trace element pollution in Antarctica is biologically relevant. Taxonomically related and/or ecologically similar species were selected for this comparison. When the same genus was not available, similar feeding strategy or same taxonomic group species were chosen. Particularly, trace element levels in SPOM were quite striking, as highlighted before. The values obtained in the present study were higher than those in the literature, except for mean Hg content compared to other Antarctic sites, similar but slightly lower than that of the SPOM of Terra Nova Bay^84^. Mean Cr content in our SPOM samples was an order of magnitude higher than that reported for the White Sea and the World Ocean. SPOM’s Pb mean level (57.57 μg g^−1^ dw) was also an order of magnitude higher than the maximum value reported previously in Antarctica and still higher than the mean value reported for the White Sea (36.05 μg g^−1^ dw), and much higher than the mean Pb value for the world ocean (8.70 μg g^−1^ dw)^85^.

**Table 3:** Trace elements concentrations (Cr, Pb, Hg) of the studied species in Antarctica.

Sample	Cr (μg g^−1^ dw)		Pb (μg g^−1^ dw)		Hg (ng g^−1^ dw)		Locality	Reference
	Range	Mean ± SD	Range	Mean ± SD	Range	Mean ± SD		
Suspended Particulate Organic Matter	1.05–32.98	10.28 ± 9.51	1.85–537.08	57.57 ± 109.48	15.12–107.26	33.49 ± 21.82	South Shetland Islands and Antarctic Peninsula	Present study
Diatom *Phaeodactylum tricornutum*	–	–	–	6.3 ± 0.20	–	–	Bellingshausen Dome, King George Island	^102^
Phytoplankton	–	–	–	5.7	–	–	Offshore waters, Maxwell Bay, King George Island	^102^
Nano and micro-phytoplankton	–	–	–	0.13 ± 0.01	–	–	Terra Nova Bay, Ross Sea	^103^
Particulate Organic Matter	–	–	–	–	28.00–47.00	39.00	Terra Nova Bay, Ross Sea	^84^
*Desmarestia anceps/ D. menziesii*	0.46–6.11	2.45 ± 1.63	0.56–4.22	1.78 ± 1.05	47.60–498.08	136.81 ± 90.57	South Shetland Islands and Antarctic Peninsula	Present study
*Desmarestia anceps*	–	–	–	–	–	32.60	Admiralty Bay, King George Island	^11^
*Desmarestia anceps*	–	3.25 ± 0.19	–	< 0.60	–	–	Potter Cove, King George Island	^71^
*Desmarestia anceps*	–	1.70 ± 0.80	–	9.40 ± 7.60	–	–	East Antarctica	^88^
*Desmarestia anceps*	–	3.25	–	0.82	–	< QLM	Ullman Point, Admiralty Bay, King George Island	^28^
*Desmarestia menziesii*	–	9.33 ± 2.69	–	< QLM	–	< QLM	Ferraz, Admiralty Bay, King George Island	^28^
*Desmarestia menziesii*	–	3	–	4.41	–	< QLM	Botany Point, Admiralty Bay, King George Island	^28^
*Palmaria decipiens*	0.14–14.41	5.39 ± 4.52	0.47–5.34	2.44 ± 4.08	10.17–1194.20	411.89 ± 330.98	South Shetland Islands and Antarctic Peninsula	Present study
*Palmaria decipiens*	–	–	–	–	–	20.40	Admiralty Bay, King George Island	^11^
*Palmaria decipiens*	–	2.05 ± 0.10	–	< 0.60	–	–	Potter Cove, King George Island	^71^
*Palmaria decipiens*	–	2.80 ± 0.30	–	2.30 ± 0.70	–	–	East Antarctica	^88^
*Nacella concinna*	0.26–3.85	1.56 ± 1.03	0.14–0.62	0.34 ± 0.14	9.66–224.55	43.55 ± 44.62	South Shetland Islands and Antarctic Peninsula	Present study
*Nacella concinna*	–	2.16 ± 0.58	–	1.42 ± 0.39	–	–	Marian Cove, King George Island	^91^
*Nacella concinna*	–	< 0.01	–	0.45 ± 0.06	–	–	Potter Cove, King George Island	^90^
*Nacella concinna*	–	–	–	–	–	26.10	Admiralty Bay, King George Island	^11^
*Nacella concinna*	–	2.57	–	< QLM	–	< QLM	Ferraz, Admiralty Bay, King George Island	^28^
*Diplasterias brucei*	0.28–1.10	0.78 ± 0.36	0.14–0.64	0.33 ± 0.13	45.92–150.13	82.95 ± 28.1	South Shetland Islands and Antarctic Peninsula	Present study
*Odontaster validus*	0.29–0.98	0.74 ± 0.29	0.21–1.68	0.55 ± 0.36	19.95–192.42	76.47 ± 47.4	South Shetland Islands and Antarctic Peninsula	Present study
*Odontaster validus* (arms)	–	0.74 ± 0.05	–	0.17 ± 0.03	–	–	Cape Evans, Ross Sea	^97^
*Odontaster validus* (arms)	–	0.70 ± 0.05	–	0.13 ± 0.03	–	–	Terra Nova Bay, Ross Sea	^97^
*Odontaster validus* (arms)	–	–	–	0.51 ± 0.22	–	–	Terra Nova Bay, Ross Sea	^104^
*Odontaster validus* (arms)	–	–	–	–	–	40.00 ± 10.00	Terra Nova Bay, Ross Sea	^101^
*Odontaster validus* (arms)	–	–	–	–	60.00–220.00	110.00	Terra Nova Bay, Ross Sea	^84^
*Odontaster validus* (integument)	–	–	–	0.60 ± 0.28	–	–	Port Foster, Deception Island	^99^

Data are given as mean ± SD, when possible. QLM: Quantification Limit of the Method.

**Table 4:** Trace elements concentrations of studied species and similar species from other regions of the world.

Sample	Cr (μg g^−1^ dw)		Pb (μg g^−1^ dw)		Hg (ng g^−1^ dw)		Locality	Reference
	Range	Mean ± SD	Range	Mean ± SD	Range	Mean ± SD		
Suspended Particulate Organic Matter	1.05–32.98	10.28 ± 9.51	1.85–537.08	57.57 ± 109.48	15.12–107.26	33.49 ± 21.82	South Shetland Islands and Antarctic Peninsula	Present study
Plankton	–	1.38	–	36.05	–	2.50	White Sea	^106^
Plankton	–	1.80	–	8.70	–	30.00	World Ocean	^85^
*Desmarestia anceps/ D. menziesii*	0.46–6.11	2.45 ± 1.63	0.56–4.22	1.78 ± 1.05	47.60–498.08	136.81 ± 90.57	South Shetland Islands and Antarctic Peninsula	Present study
*Desmarestia aculeata*	0.56–2.40	–	0.08–1.60	––	–	–	Spitsbergen, Svalbard Islands	^113^
*Padina tenuis*	1.40–10.00	–	0.10–6.20	–	–	–	Townsville coastal waters, Queensland, Australia	^114^
*Padina tetrastromatica*	1.60–9.90	–	1.10–10.20	–	–	–	Townsville coastal waters, Queensland, Australia	^114^
*Nizamuddinia zanardinii*	–	–	–	0.17	–	6.00	Dhofar, southern Oman (relatively unspoiled)	^89^
*Fucus serratus*	0.70–2.60	–	4.00–21.00	–	–	–	Scotland	^72^
*Stypocaulon scoparium*	–	–	–	–	100.00–200.00	–	Port-Cros Bay, France	^115^
*Stypocaulon scoparium*	–	–	–	–	–	30.00	Port-Cros National Park, France	^115^
*Palmaria decipiens*	0.14–14.41	5.39 ± 4.52	0.47–5.34	2.44 ± 4.08	10.17–1194.20	411.89 ± 330.98	South Shetland Islands and Antarctic Peninsula	Present study
*Palmaria palmata*	–	0.16 ± 0.15	–	0.04 ± 0.00	–	–	Asia (China, Japan, South Korea)	^86^
*Palmaria palmata*	–	0.08 ± 0.05	–	0.05 ± 0.03	–	–	European Union	^86^
*Palmaria palmata*	–	–	–	4.40 ± 0.30	–	–	Purchased in Italy	^87^
*Gracilaria longissima*		0.90 ± 0.10	–	0.90 ± 0.40	–	–	Gulf of Kutch, India	^116^
*Gelidium* sp.	–	–	–	1.41	–	8.00	Dhofar, southern Oman (relatively unspoiled)	^89^
*Nacella concinna*	0.26–3.85	1.56 ± 1.03	0.14–0.62	0.34 ± 0.14	9.66–224.55	43.55 ± 44.62	South Shetland Islands and Antarctic Peninsula	Present study
*Patella caerulea*	–	–	0.67–1.29	0.98	–	–	Spanish Mediterranean coast	^117^
*Cellana rota*	–	–	–	0.45	–	21.00	Dhofar, southern Oman (relatively unspoiled)	^89^
*Diplasterias brucei*	0.28–1.10	0.78 ± 0.36	0.14–0.64	0.33 ± 0.13	45.92–150.13	82.95 ± 28.1	South Shetland Islands and Antarctic Peninsula	Present study
*Odontaster validus*	0.29–0.98	0.74 ± 0.29	0.21–1.68	0.55 ± 0.36	19.95–192.42	76.47 ± 47.4	South Shetland Islands and Antarctic Peninsula	Present study
*Echinaster sepositus*	–	–	–	–	930.00–1620.00	–	Port-Cros Bay, France	^115^
*Echinaster sepositus*	–	–	–	–	–	100.00	Port-Cros National Park	^115^
*Echinaster sepositus*	–	0.83	–	–	–	–	Saronikos gulf, Greece	^98^
*Marthasterias glacialis*	–	–	–	–	–	210.00	Port-Cros Bay, France	^115^
*Marthasterias glacialis*	–	1.64	–	–	–	–	Saronikos gulf, Greece	^98^
*Astropecten aurentiacus*	–	–	–	–	–	80.00	Port-Cros Bay, France	^115^
*Asterias rubens* (oral body wall)	–	–	–	0.85 ± 0.63	–	–	Belgian coast (polluted)	^100^
*Asterias rubens* (aboral body wall)	–	–	–	0.36 ± 0.19	–	–	Belgian coast (polluted)	^100^

Data are given as mean ± SD, when possible.

Regarding the red algae *Palmaria decipiens*, the mean Cr content doubled the recorded in other works and the range obtained in this study was broad (Table 4). Pb content in the specimens collected in this study are from one to two orders of magnitude higher than samples of edible red algae *Palmaria palmata* cultivated in Asia and in the European Union^86^, and with a lower mean value, even though still within the same range of values, than those of *Palmaria palmata* sold in Italy^87^. The mean Pb content in the Antarctic red algae was higher than that of two other different Rodophyceae collected in India and Oman, the latter considered a relatively not impacted place (Table 4).

For Cr, the mean content in *Desmarestia* spp. (2.4 μg g^−1^ dw) fell between the mean value in Runcie and Riddle^88^ (1.70 μg g^−1^ dw) and that in Farías et al.^71^ (3.25 μg g^−1^ dw), and was slightly lower than that reported in Trevizani et al.^28^ near Comandante Ferraz station, in King Geoge Island (South Shetland Islands) (9.33 μg g^−1^ dw). Cr range values in *Desmarestia* spp. and other Phaeophyceae from such different sites as Australia, Svalbard Islands, and Scotland fell within the same range (Table 4). Regarding Pb, the range of values in *Desmarestia* anceps and *Desmarestia menziesii* was similar to that of *Desmarestia aculeata* collected in the Svalbard Islands and *Padina tenuis*, another brown algae collected in Australian waters (Table 4). The mean Pb content in *Desmarestia* spp. from the present study was ten times higher than that of the brown algae *Nizamuddinia zanardinii* from a site in southern Oman^89^, suggesting the South Shetland Islands and the Antarctic Peninsula are impacted by anthropogenic pollution. On the other hand, the values measured in other brown algae were higher than those presented in this study. The maximum value in *Padina tetrastromatica* from Australia more than doubles the highest concentration in Antarctic *Desmarestia* spp. and *Fucus serratus*. Pb minimum value was similar to the highest value obtained in this study.

The Cr mean value for *Nacella concinna* fell between the mean values reported in Potter Cove^90^ and Admiralty Bay^28^, both in King George Island (South Shetland Islands), even though the maximum level obtained in our study exceeded the highest value reported in the literature. Pb mean level in *Nacella concinna* was slightly lower than that reported for the same species in Potter Cove^90^ and also fell below that reported in Marian Cove (King George Island)^91^ (Table 3). Other limpet species in Southern Oman and the Spanish Mediterranean coast had slightly higher levels of Pb than those reported in the present study (Table 4), although the maximum value here is higher than the average there. Hg mean value reported here for *Nacella concinna* almost doubles that reported for the same species in King George Island and that reported for the limpet *Cellana rota* from southern Oman.

There were no previous data for Cr, Pb, and Hg levels in *Diplasterias brucei* in the literature. Both asteroids, *Diplasterias brucei* and *Odontaster validus*, are carnivores consuming other invertebrates, such as molluscs, crustaceans, ostracods, and sponges. *Odontaster validus* is known to exploit further feeding modes^92–96^. The concentrations of trace elements were of the same order of magnitude in both starfish species. Both species had similar Cr content than that measured in *Odontaster validus* in other studies from two areas in the Ross Sea^97^. Cr mean content in both starfish was similar to the Cr content in *Echinaster sepositus* from Saronikos gulf, Greece, and half the mean value of *Marthasterias glacialis* collected in the same site^98^. Pb values reported in the present study for the starfishes were more than twice the Pb concentration in *Odontaster validus* reported by Grotti et al.^97^ (Table 3) and were very similar to those reported for the same species in Deception Island (South Shetland Islands)^99^ and for the common starfish *Asterias rubens* in the Belgian coast^100^. The Hg mean levels reported in our study fell between the levels reported for *Odontaster validus* of Terra Nova Bay in Dalla Riva et al.^101^ (40.00 ng g^−1^) and Bargagli et al.^84^ (110.00 ng g^−1^ dw). Hg concentrations were the same as those of *Astropecten aurentiacus* in Port-Cros Bay (France), but lower than those measured in *Echinaster sepositus* in Port-Cros National Park (France), where low pollution is assumed, and *Marthasterias glacialis* in Port-Cros Bay (France) (Table 4).

In general, Cr, Pb, and Hg levels obtained here fell in the range of or were higher than those measured in other sites around Antarctica, which indicates the reliability of the results in this study. The Cr levels in SPOM and *Palmaria decipiens* levels were around 2 to 10 times higher than those of related species, suggesting trace element levels in Antarctic biota are not negligible, but rather concerning. Regarding Pb, it seems to act as an indicator of the human presence in Antarctica. The mean values obtained in the present study for the primary producers were higher than those recorded for primary producers at three different sites in King George Island, which Curtosi et al.^90^ declared practically unaffected by pollution, and Terra Nova Bay in the Ross Sea, more remote and less exposed to human local activity than the Antarctic Peninsula^84^. In Terra Nova Bay, however, there are several research facilities, namely the stations McMurdo, Scott Base, Mario Zucchelli, Gondwana, and Jang Bogo^56^ and previous reports of contamination exist^105^. For the three animal species, values of Pb are either similar or slightly lower than those measured in ecologically comparable species from other coastal regions of the world. In general, Hg levels in our studied species were higher than those reported for the same species in the literature and for other species from other regions of the world (Tables 3 and 4). Specifically, all the Hg concentrations measured by Santos et al.^11^ in Admiralty Bay, King George Island, were lower than those reported in the present study. Santos et al.^11^ reported very low levels of trace elements, i.e. close to natural levels. The Hg value reported for the World Ocean was reported to be doubtful in Demina and Nemirovskaya^106^ since it is an order of magnitude higher than that measured in the White Sea in the mentioned study. The authors attributed this to the utilization of different procedures for sample preparation and analyses.

Although there is a large diversity of macroalgae in shallow rocky bottoms around Antarctica^57,107^, they are minor contributors to the carbon pool fueling the food web^50,51,54^ because most of them are chemically defended from herbivores through phlorotannins and other natural products^96,108–112^. This may explain why the levels of trace elements in limpets and starfish are much lower than in macroalgae, assuming that they lack mechanisms to remove these trace elements, i.e. detoxification. Phytoplankton is often considered to be the primary source of carbon for shallow Antarctic food webs^50–54^. The levels of Hg in SPOM reported in this study are consistent with this hypothesis, as they are lower than those observed in consumers from the same site. However, Pb levels in SPOM were much higher than in limpets and starfish. As Pb is known to biomagnify, these results may suggest limited reliance of the three consumers on SPOM. However, SPOM could be a relevant food source if the Pb trapped in diatom frustules^79^ is not absorbed by consumers relying on SPOM. Nevertheless, previous research based on stable isotopes of C has shown that encrusting corallinaceous algae are a major dietary item for limpets^55^, but unfortunately, we had not enough material to analyze Hg levels in encrusting algae. In any case, the Hg content of the two starfish was higher than that of the limpet, a result consistent with their higher trophic position^54^ and demonstrative of the Hg biomagnification along Antarctic food webs.

## Conclusions

A latitudinal Cr, Pb and Hg pollution gradient along the South Shetland Islands and the Antarctic Peninsula was not observed in the studied representative benthic species, possibly because the sampling sites were sheltered coastal zones more influenced by local factors than by processes operating at broader geographic scales. This gradient may still exist, however, and further research is needed to prove it.

Differences in concentration among sites and species were found for the three trace elements studied. Nevertheless, the existence of more globally polluted sites could not be demonstrated, as each species responded independently at each sampling site. The primary producers included in this study have a higher trace element content than the selected animal species, suggesting that neither the particulate organic matter nor the dominant macroalgae in this community are the food web carbon source. This supports our previous results on trophic analysis of this community, revealing encrusting corallinaceous algae as the most likely carbon source for the limpet *Nacella concinna*. In agreement with previous research, the starfishes *Odontaster* validus and *Diplasterias brucei*, top predators in the community, may be feeding on the limpet *Nacella concinna*.

In general, Cr, Pb and Hg levels fell in the range of or were higher than those measured in other Antarctic places. Remarkably, trace element concentrations reported in this study are in general comparable to those of taxonomically or ecologically similar species from other coastal regions of the world, thus indicating that at least the Antarctic Peninsula and South Shetland Islands are not so pristine and unspoiled as it may be generally considered but they are as polluted as the rest of the world. Therefore, monitoring pollution levels in these regions is an important and urgent task to be done.

## Supplementary Information

Supplementary Information.

## Data Availability

The datasets generated and analyzed during the current study are available from the corresponding author on reasonable request.
