# Supplementary material for: Distribution of trace elements in benthic infralittoral organisms from the western Antarctic Peninsula reveals no latitudinal gradient of pollution
Source: Sci Rep. 2021 Aug 11;11:16266. doi: 10.1038/s41598-021-95681-5 (PMC8357953; doi:10.1038/s41598-021-95681-5)
Supplement: Supplementary file 1 — Supplementary Information. [file 41598_2021_95681_MOESM1_ESM.pdf]

SUPPLEMENTARY INFORMATION

Distribution of trace elements in benthic infralittoral organisms from the western Antarctic Peninsula reveals no latitudinal gradient of pollution

Paula de Castro-Fernández*, Luis Cardona & Conxita Avila

Department of Evolutionary Biology, Ecology and Environmental Sciences & Biodiversity Research Institute (IRBio), University of Barcelona, Diagonal Ave. 643, 08028, Barcelona, Catalonia.

*Corresponding author. E-mail address: p.decastro@ub.edu

Supplementary table 1

Range and mean values ± SD of Cr, Pb, and Hg in the studied species at each sampling site.

Species	Cr (µg g^{-1} dw)		Pb (µg g^{-1} dw)		Hg (ng g^{-1} dw)	
	Range	Mean ± SD	Range	Mean ± SD	Range	Mean ± SD
**Suspended Particulate Organic Matter (SPOM)**						
Fildes Bay	3.49-25.33	12.46 ± 8.41	10.53-89.61	30.55 ± 33.23	21.43-43.14	30.91 ± 9.53
Hope Bay	6.18-32.87	14.51 ± 10.6	15.73-117.79	48.07 ± 40.13	29.58-46.2	35.93 ± 7.26
Cierva Cove	1.07-8.8	3.25 ± 3.19	1.85-30.81	9.75 ± 12.04	15.12-16.64	15.68 ± 0.71
Paradise Bay	1.05-32.98	8.11 ± 13.92	2.12-120.04	27.66 ± 51.68	15.4-21.96	18.63 ± 3.01
Rothera Point	7.25-24.7	13.07 ± 7.07	14.34-537.08	171.87 ± 212.28	37.23-107.26	66.31 ± 25.88
Desmarestia anceps/ D. menziesii						
Fildes Bay	1.23-4.63	2.36 ± 1.41	0.56-1.71	1.05 ± 0.5	47.6-140.06	94.97 ± 37.36
Hope Bay	0.46-4.23	1.91 ± 1.41	0.56-1.26	0.87 ± 0.29	56.62-498.08	184.69 ± 183.93
Cierva Cove	1.62-2.63	2.28 ± 0.4	2.21-4.22	3.35 ± 0.74	104.97-153.39	120.43 ± 19.78
Paradise Bay	4.22-6.11	4.85 ± 0.9	1.62-3.41	2.17 ± 0.73	70.94-294.34	164.46 ± 80.88
Rothera Point	0.47-1.41	0.85 ± 0.39	1.18-1.79	1.46 ± 0.26	93.24-137.04	119.48 ± 18.3
Palmaria decipiens						
Fildes Bay	2.31-3.81	3.12 ± 0.68	0.47-0.99	0.76 ± 0.23	307.35-1123.31	645.41 ± 354.88
Hope Bay	6.05-14	10.04 ± 2.94	1.50-1.89	1.7 ± 0.14	531.99-718.65	620.83 ± 67.2
Cierva Cove	0.14-8.16	2.63 ± 3.56	0.51-3.24	1.49 ± 1.2	10.17-175.51	70.16 ± 75.63
Paradise Bay	0.8-4.04	2.13 ± 1.18	2.27-3.76	2.93 ± 0.64	240.7-577.29	382.28 ± 123.52
Rothera Point	2.87-14.41	9.05 ± 5.14	0.87-21.49	5.34 ± 9.05	58.1-1194.2	340.79 ± 479.3
Nacella concinna						
Fildes Bay	0.66-1.35	1.03 ± 0.28	0.14-0.22	0.18 ± 0.03	23.09-43.31	30.64 ± 7.88
Hope Bay	1.88-3.5	2.62 ± 0.72	0.23-0.42	0.33 ± 0.09	34.2-75.75	55.08 ± 17.02
Cierva Cove	0.92-3.85	1.85 ± 1.22	0.15-0.43	0.26 ± 0.1	23.57-224.55	86.04 ± 84.03
Paradise Bay	0.26-0.62	0.45 ± 0.16	0.42-0.62	0.51 ± 0.08	9.66-16.23	11.9 ± 2.65
Rothera Point	1.25-3.28	1.84 ± 0.85	0.27-0.48	0.41 ± 0.09	17.89-72.12	34.09 ± 23.05
Diplasterias brucei						
Fildes Bay	0.61-1.41	0.94 ± 0.33	0.14-0.28	0.19 ± 0.06	47.99-87.37	67.26 ± 14.93
Hope Bay	0.53-1.39	0.95 ± 0.37	0.2-0.34	0.27 ± 0.05	71.88-141.91	103.44 ± 25.33
Cierva Cove	0.72-1.39	1.1 ± 0.28	0.25-0.45	0.33 ± 0.07	77.25-150.13	111.84 ± 28.86
Paradise Bay	0.48-0.54	0.51 ± 0.03	0.43-0.64	0.53 ± 0.07	57.84-90.18	74.81 ± 11.73
Rothera Point	0.28-0.51	0.41 ± 0.11	0.23-0.37	0.29 ± 0.05	45.92-70.19	57.39 ± 11.27
Odontaster validus						
Fildes Bay	0.5-0.92	0.74 ± 0.16	0.23-0.32	0.25 ± 0.04	33.74-80.79	61.87 ± 17.76
Hope Bay	0.76-1.31	0.98 ± 0.21	0.21-0.91	0.38 ± 0.3	63.97-192.42	123.79 ± 49.26
Cierva Cove	0.3-1.09	0.59 ± 0.3	0.46-1.68	0.91 ± 0.49	70.79-184.07	112.63 ± 44.9
Paradise Bay	0.29-0.54	0.43 ± 0.1	0.61-1.02	0.72 ± 0.17	19.95-45.28	25.79 ± 10.94
Rothera Point	0.73-1.27	0.95 ± 0.2	0.32-0.91	0.48 ± 0.24	37.19-78.29	58.26 ± 16.75

Supplementary table 2
Results of the general lineal model used to assess the effect of species and site on standardized Cr levels.

Tests of Between-Subjects Effects					
Dependent Variable: **Cr**					
Source	Type III Sum of Squares	df	Mean Square	F	Sig.
Corrected Model	83.294[a]	29	2.872	5.246	< 0.001
Intercept	0.000	1	0.000	0.000	1.000
species	57.583	5	11.517	21.033	< 0.001
site	6.466	4	1.617	2.952	0.023
species * site	19.245	20	0.962	1.757	0.033
Error	65.706	120	0.548		
Total	149.000	150			
Corrected Total	149.000	149			

a. R Squared = 0.559 (Adjusted R Squared = 0.452)

Supplementary table 3
Results of the general lineal model used to assess the effect of species and site on standardized Pb levels.

Tests of Between-Subjects Effects					
Dependent Variable: **Pb**					
Source	Type III Sum of Squares	df	Mean Square	F	Sig.
Corrected Model	63.824[a]	29	2.201	3.101	< 0.001
Intercept	0.000	1	0.000	.000	1.000
species	27.963	5	5.593	7.879	< 0.001
site	6.191	4	1.548	2.180	0.075
species * site	29.670	20	1.483	2.090	0.008
Error	85.176	120	0.710		
Total	149.000	150			
Corrected Total	149.000	149			

a. R Squared = 0.428 (Adjusted R Squared = 0.290)

Supplementary table 4

Results of the general lineal model used to assess the effect of species and site on standardized Hg levels.

Tests of Between-Subjects Effects					
Dependent Variable: **Hg**					
Source	Type III Sum of Squares	df	Mean Square	F	Sig.
Corrected Model	101.303[a]	29	3.493	8.788	< 0.001
Intercept	0.000	1	0.000	0.000	1.000
species	68.752	5	13.750	34.594	< 0.001
site	5.230	4	1.307	3.289	0.013
species * site	27.322	20	1.366	3.437	<0.001
Error	47.697	120	.397		
Total	149.000	150			
Corrected Total	149.000	149			

a. R Squared = 0.680 (Adjusted R Squared = 0.603)

Supplementary table 5

Results of the general lineal model used to assess the effect of species and site on standardized Cr levels for the three animal species (*N. concinna*, *D. brucei*, *O. validus*).

Tests of Between-Subjects Effects					
Dependent Variable: **Cr**					
Source	Type III Sum of Squares	df	Mean Square	F	Sig.
Corrected Model	49.671a	14	3.548	8.750	<0.001
Intercept	0.000	1	0.000	0.000	1.000
species	19.210	2	9.605	23.688	<0.001
site	16.096	4	4.024	9.924	<0.001
species * site	14.365	8	1.796	4.429	<0.001
Error	24.329	60	0.405		
Total	74.000	75			
Corrected Total	74.000	74			

a. R Squared = 0.671 (Adjusted R Squared = 0.595)

Supplementary table 6

Results of the general lineal model used to assess the effect of species and site on standardized Pb levels for the three animal species (*N. concinna*, *D. brucei*, *O. validus*).

Tests of Between-Subjects Effects					
Dependent Variable: **Pb**					
Source	Type III Sum of Squares	df	Mean Square	F	Sig.
CorrectedModel	44.465a	14	3.176	6.452	<0.001
Intercept	0.000	1	0.000	0.000	1.000
species	12.349	2	6.175	12.544	<0.001
site	20.364	4	5.091	10.342	<0.001
species * site	11.752	8	1.469	2.984	0.007
Error	29.535	60	0.492		
Total	74.000	75			
Corrected Total	74.000	74			

a. R Squared = 0.601 (Adjusted R Squared = 0.508)

Supplementary table 7

Results of the general lineal model used to assess the effect of species and site on standardized Hg levels for the three animal species (*N. concinna*, *D. brucei*, *O. validus*).

Tests of Between-Subjects Effects					
Dependent Variable: **Hg**					
Source	Type III Sum of Squares	df	Mean Square	F	Sig.
CorrectedModel	42.799a	14	3.057	5.879	<0.001
Intercept	0.000	1	0.000	.000	1.000
species	11.549	2	5.775	11.105	<0.001
site	26.520	4	6.630	12.749	<0.001
species * site	4.730	8	0.591	1.137	0.352
Error	31.201	60	0.520		
Total	74.000	75			
Corrected Total	74.000	74			

a. R Squared = 0.578 (Adjusted R Squared = 0.480)

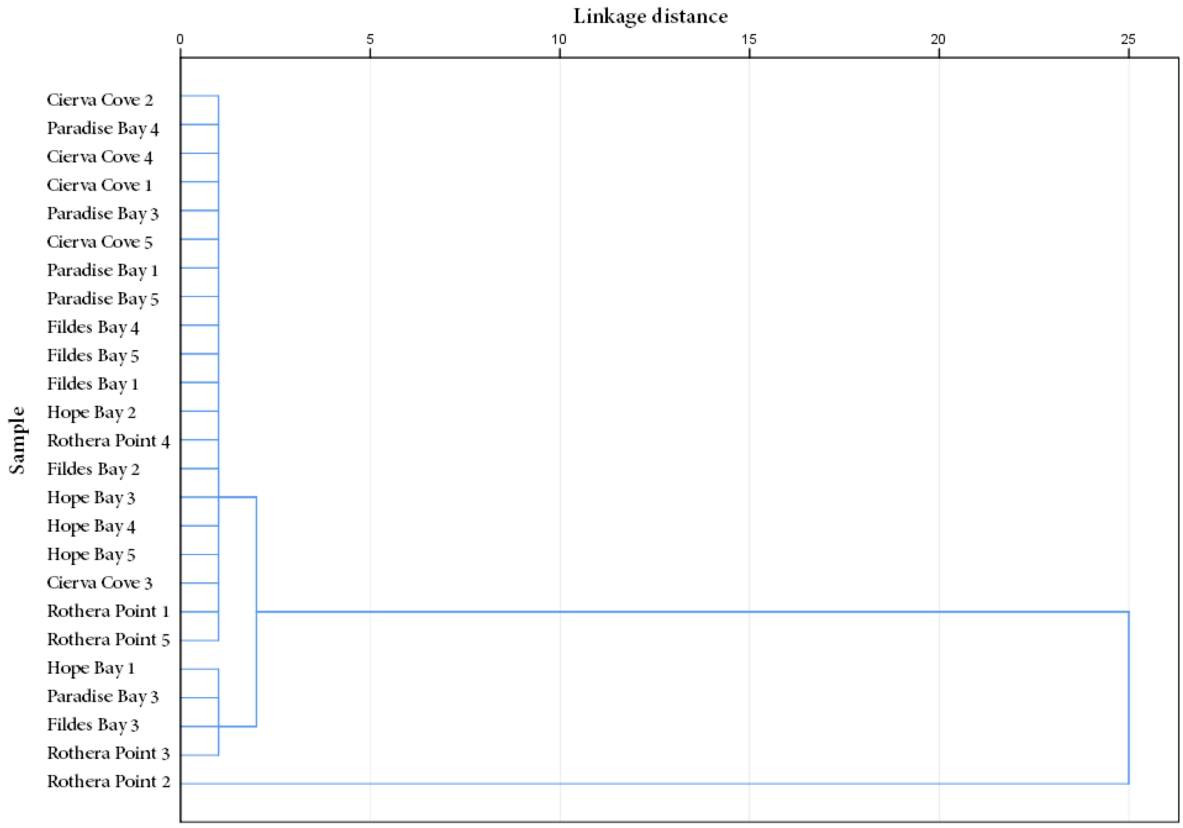

Supplementary figure 1. Dendrogram for SPOM samples obtained using the squared Euclidean distance as a metric and UPGMA as the clustering method.

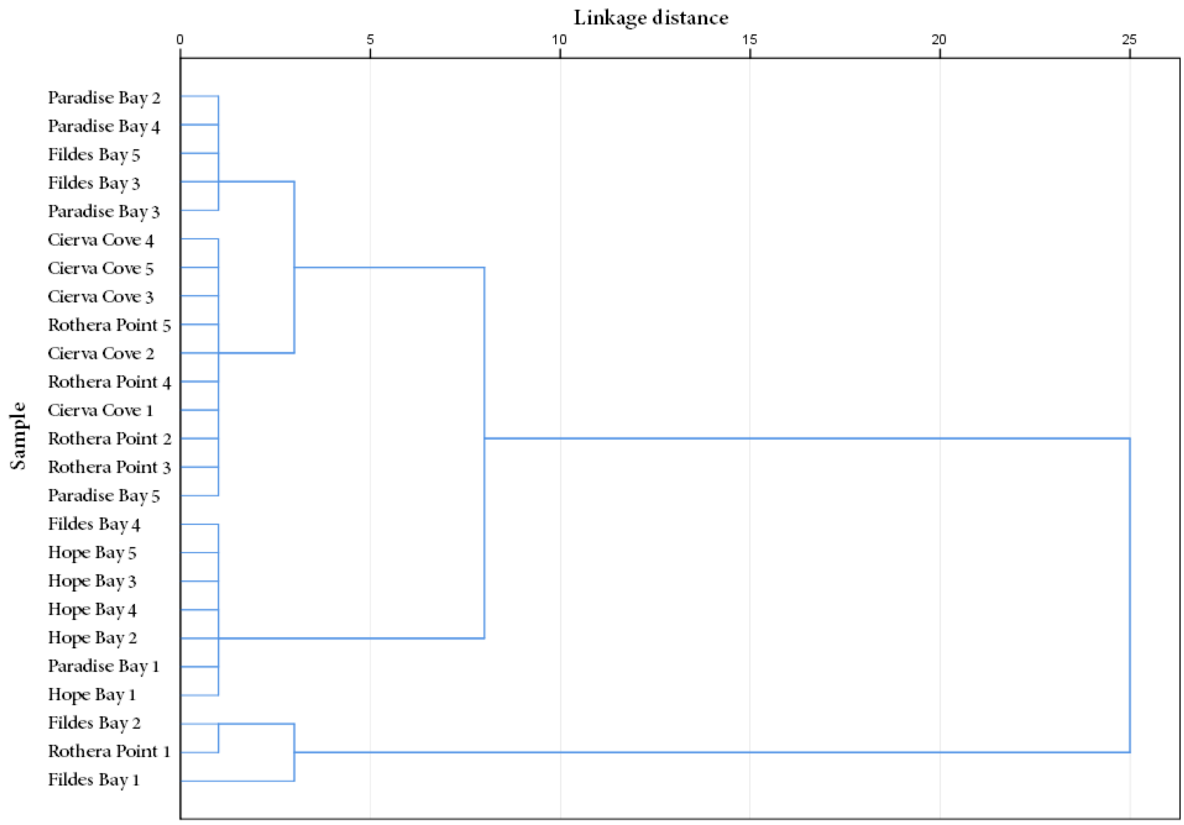

Supplementary figure 2. Dendrogram for *Palmaria decipiens* samples obtained using the squared Euclidean distance as a metric and UPGMA as the clustering method.

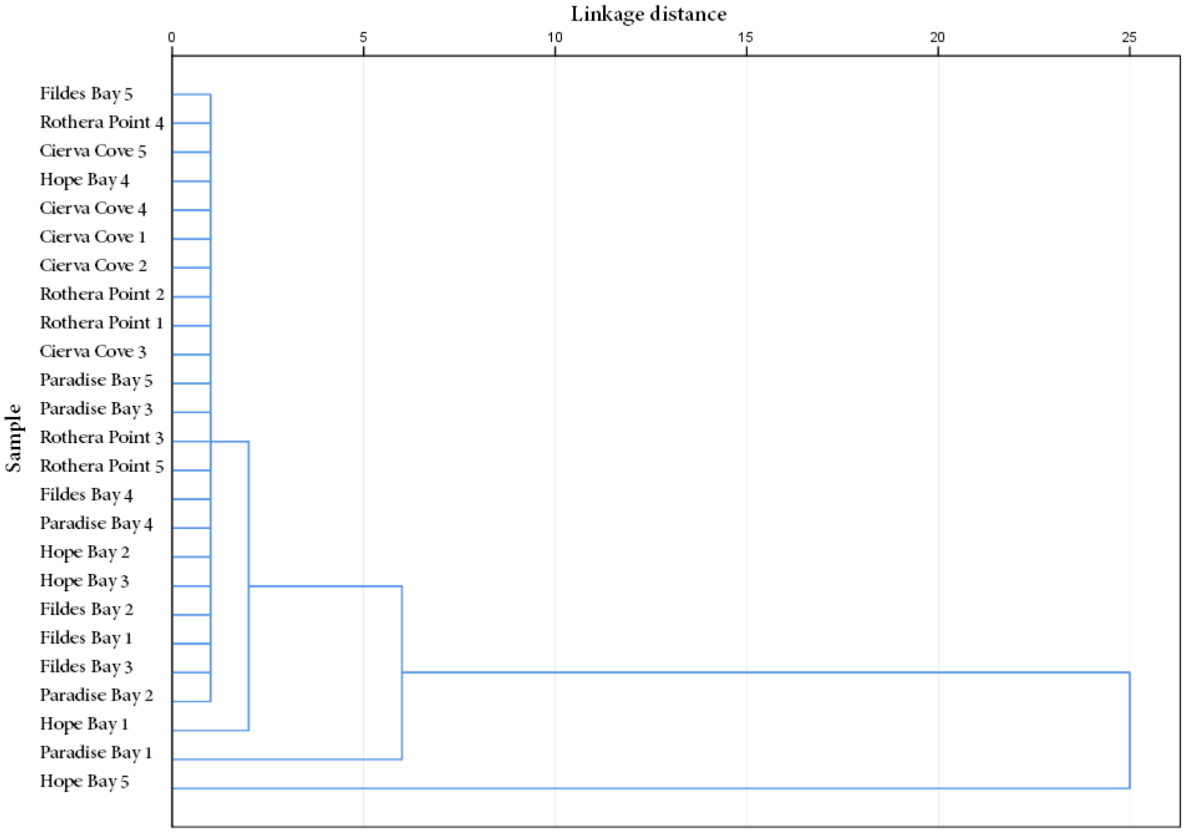

Supplementary figure 3. Dendrogram for *Desmarestia* sp. samples obtained using the squared Euclidean distance as a metric and UPGMA as the clustering method.

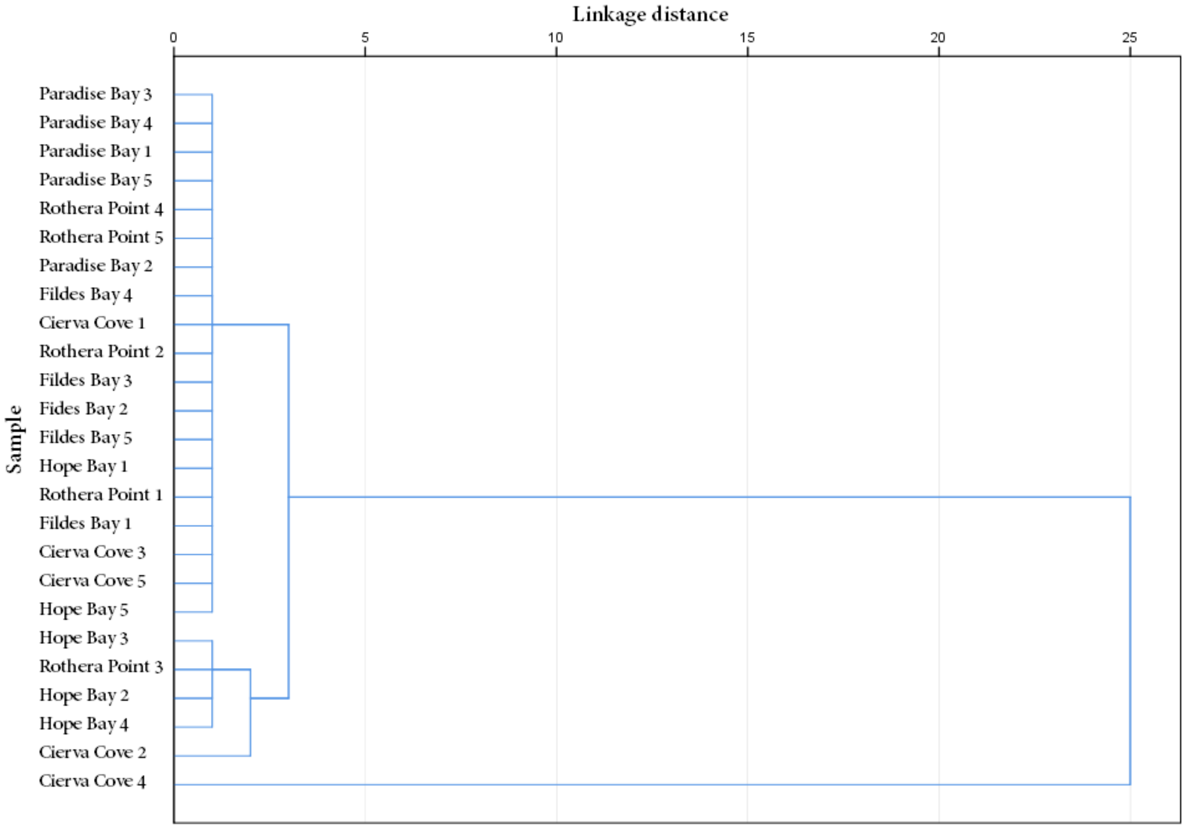

Supplementary figure 4. Dendrogram for *Nacella concinna* samples obtained using the squared Euclidean distance as a metric and UPGMA as the clustering method.

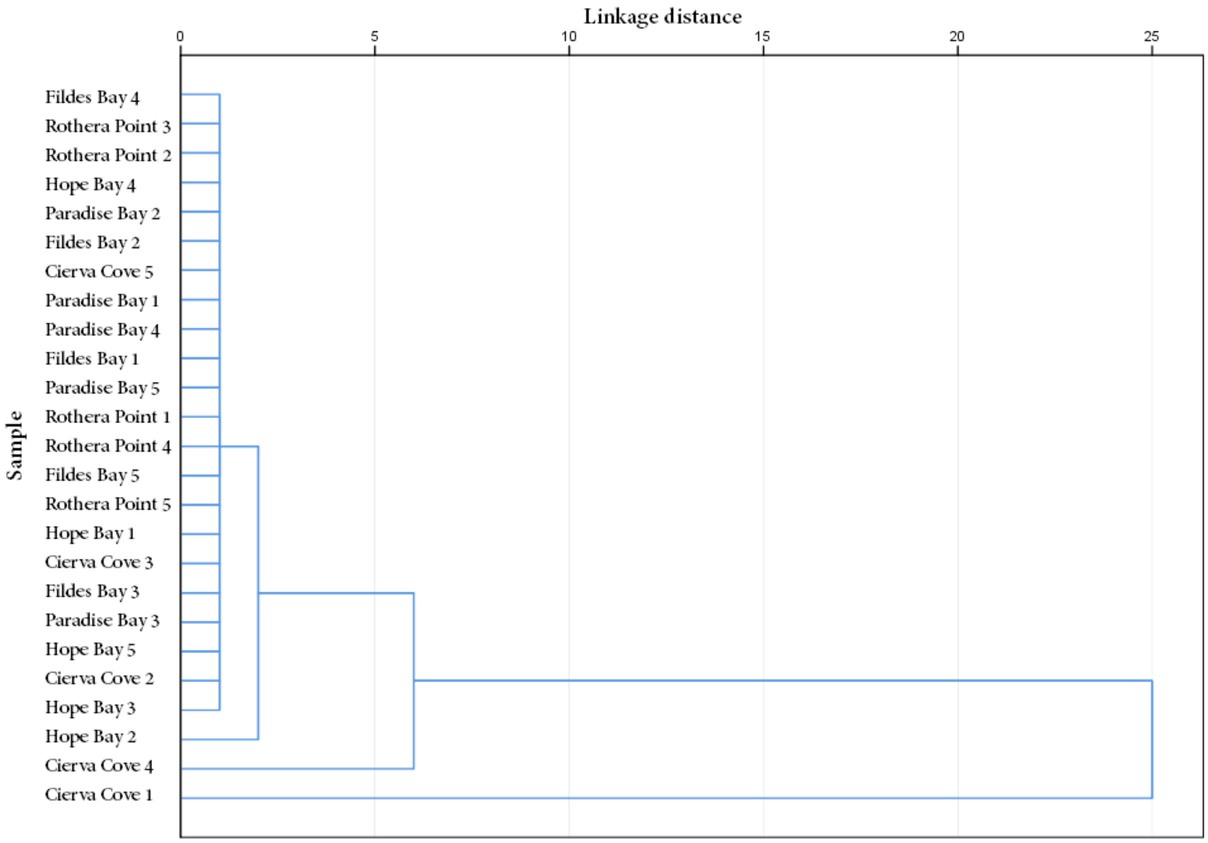

Supplementary figure 5. Dendrogram for *Diplasterias brucei* samples obtained using the squared Euclidean distance as a metric and UPGMA as the clustering method.

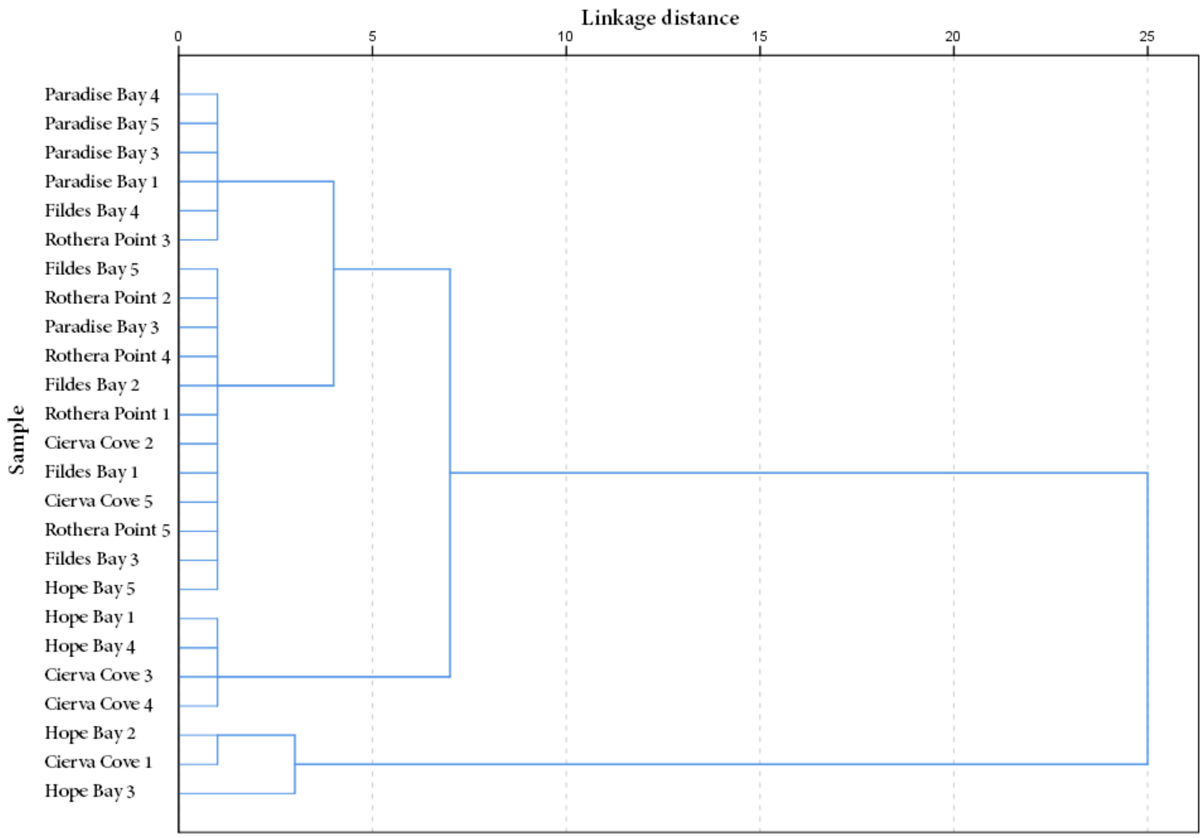

Supplementary figure 6. Dendrogram for *Odontaster validus* samples obtained using the squared Euclidean distance as a metric and UPGMA as the clustering method.
